# Long term clinical results of intrabony defects treated with periodontal regeneration. A retrospective analysis based on composite outcome measure

**DOI:** 10.1007/s00784-025-06633-6

**Published:** 2025-11-15

**Authors:** Andrea Blasi, Leopoldo Mauriello, Vincenzo Iorio-Siciliano, Vitolante Pezzella, Gaetano Isola, Luca Ramaglia

**Affiliations:** 1 Department of Periodontology, School of Dental Medicine, University of Naples Federico II, Via S. Pansini 5, 80131 Naples, Italy; 2 Unit of Periodontology, Department of General Surgery and Medical Surgical Specialties, University of Catania, 95124 Catania, Italy

**Keywords:** Periodontitis, Regeneration, Treatment outcome, Attachment loss, Periodontal pocket, Bone graft

## Abstract

**Objectives:**

To report the long-term results of intrabony defects following periodontal regenerative therapy up to 7-years using composite outcome measure (COM).

**Materials and methods:**

Clinical charts of 19 patients with 29 periodontal intrabony defects treated using periodontal regeneration were selected for the analysis. The intrabony defects were treated using different flap designs (extended flap with simplified papilla preservation technique, minimally invasive surgical technique or modified minimally invasive surgical technique) and different regenerative treatments (guided tissue regeneration, enamel matrix derivative or deproteinized bovine bone mineral combined with enamel matrix derivative) were applied. Full-mouth plaque score (FMPS, full-mouth bleeding score (FMBS), clinical attachment level (CAL), probing depth (PD), and gingival recession (GM) were recorded at baseline, at 1, and 7 years. The effects of periodontal regeneration were evaluated according to COM values at 1 and 7 years. Intrabony defects were classified as COM1 (i.e. CAL gain ≥ 3 mm, PD ≤ 4 mm), COM2 (i.e. CAL gain < 3 mm, PD ≤ 4 mm), COM3 (i.e. CAL gain ≥ 3 mm, PD > 4 mm), COM4 (i.e. CAL gain < 3 mm, PD > 4 mm). The primary outcome was COM change. Treatment success was defined as a site displaying a CAL gain ≥ 3 mm and a PD ≤ 4 mm (COM 1).

**Results:**

After 7 years, a statistically significant improvement was observed in all clinical parameters (*p* < 0.05). At 1- and 7-years follow-up, the proportions of defects considered COM1 (i.e. treatment success) were 75.9% and 44.8%, respectively. After 1 year following regenerative therapy, 3.4% of the defects were defined as COM2 and COM3, while 17.2% of the sites was assigned to COM4. At 7 years, the proportion of defects in COM2 and COM4 was 27.6% in each group, whereas no defects showed a COM3 value.

**Conclusions:**

Within limits of the present study, the results indicated that the clinical benefits obtained following regenerative therapy can be maintained on a period of 7 years. However, the treatment success (i.e. COM1) was not always achieved.

**Clinical relevance:**

Despite a relevant CAL gain (i.e. CAL gain ≥ 3 mm), the presence of residual pockets (i.e. PD > 4 mm) following periodontal regeneration could determine a worsening of clinical parameters during supportive periodontal care.

## Introduction

Regenerative therapy is considered an effective and predictable treatment in the management of intrabony defects resulting in improved clinical outcomes (i.e. clinical attachment gain, probing depth reduction and tooth survival rate) when compared with access flap alone [1, 2].

The success of regenerative treatments is influenced by several factors such as the morphology of the defect (i.e., number of residual bone walls), the surgical technique (i.e. access flap with papilla preservation or minimally invasive surgical techniques), the regeneration procedures (i.e., guided tissue regeneration or application of biologic agents) and other factors related to the patient (i.e., smoking habit and plaque control) [3].

In the last decades, many clinical approaches [4–7] were proposed to treat the intrabony defects (i.e. membranes or biologic agents with or without the addition of bone-derived grafts), and the effectiveness of these procedures was confirmed by histological findings in human [8].

Although the histological analyses represent the definitive proof to verify the outcomes of periodontal regeneration, these examinations are not realistic outcome measures for clinical trials and daily practice. For these reasons the efficacy of regenerative procedures is tested using clinical parameters, and usually, the CAL gain is set as primary endpoint [9]. Data of systematic reviews reported that at least 3 mm of CAL gain were considered clinically relevant to detect the efficacy of regenerative procedure [10–12]. Available evidence demonstrated that the CAL gain (i.e., at least 3 mm) achieved at 1 year follow-up following regenerative therapy can be maintained over time for more than 10 years [13, 14], and regenerated intrabony defects showed lower probability for recurrence of pockets and lower costs of reintervention than sites treated with access flap alone over a 20-years follow-up period [15].

However, the stability of intrabony defects treated by means of regenerative procedures depends not only on the CAL gain ≥ 3 mm but also on the pocket closure (i.e. PD ≤ 4 mm without bleeding on probing), since residual pockets (i.e. PD ≥ 5 mm) represent a risk factor for disease progression and tooth loss [16]. Moreover, the World Workshop on Periodontal Diseases and Conditions considered PD ≤ 4 mm on all sites the pre-requisite for the stability of successfully treated periodontitis patients [17].

Hence, the success and stability of intrabony defects following regenerative therapies should be assessed using a combination of both endpoints (i.e. CAL gain and PD reduction) included in a composite outcome measure (COM). COM showed better accuracy in verifying the stability of intrabony defects treated using regenerative procedures than single probing measurements (i.e. CAL gain and PD reduction analyzed separately), and they also offered the possibility to better identify the failures of regenerative therapy [18].

Nowadays, composite outcome measures were proposed to verify the short-term results of periodontal regenerative therapy [19], and data on the prognostic value of COM for the long-term stability following regenerative therapy are limited [20]. Hence, the aim of this study was to evaluate the outcomes of intrabony defects treated with periodontal regenerative therapy using COM values after 7 years follow-up.

## Material and methods

### Study design

The present study was designed as retrospective clinical analysis with 7 years of follow-up. The clinical charts of patients affected by periodontitis with at least one intrabony defects treated using a regenerative approach were screened. The screening of clinical chart was performed from January 2017 to December 2024.

The analysis involved a retrospective analysis of pre-existing data. Therefore, no experimental procedures were performed, however, the study was approved by the Institutional Review Board of University of Catania (approval number: prot.310).

The study was conducted according to the principles of the Declaration of Helsinki on experimentation involving human subjects. Written consent was obtained from all participants before the study.

### Patient population

All patients were treated at Department of Periodontology, University of Naples Federico II and enrolled in supportive periodontal care (SPC). Collected data were analyzed in the Department of Periodontology, University of Catania (Italy).

The investigation used a clinical database including patients who received a periodontal regeneration therapy as a part of routine periodontal care using accepted therapy for each patient’s specific clinical needs.

### Eligibility criteria

To be included in the study, the following inclusion criteria had to be fulfilled:

- Patients of either gender with diagnosis of periodontitis Stage III or Stage IV [21].
- Age ≥ 18 years
- Availability of periodontal chart at time of periodontal regenerative surgery and at 7-year follow-up period.
- Patients with at least one periodontal intrabony defect with a probing depth (PD) ≥ 6 mm associated to an intrabony defects ≥ 3 mm.
- Intrabony defects treated by means of guided tissue regeneration (i.e. resorbable membrane and deproteinized bovine bone mineral; DBBM), or enamel matrix derivative (EMD), or EMD combined with DBBM.
- Single rooted or multi-rooted teeth in either maxilla or mandible.
- Patients enrolled in a regular supportive periodontal care (SPC) program based on a combination of preventive and therapeutic interventions (i.e., monitoring of periodontal health, reinforcement of oral hygiene instructions, patient motivation and professional mechanical plaque removal) rendered at different personalized intervals [22] for a period at least 7 years.

The exclusion criteria were as follows:

- Patients with systemic diseases contraindicating periodontal surgery.
- Tobacco smokers (≥ 10 cigarettes/d).
- Supra-bony defects
- Multi-rooted teeth with class II and class III furcation defects.
- Erratic adherence to the SPC program.

### Outcome measures

The following outcome measures were recorded:

- Full-Mouth Plaque Score (FMPS), representing the percentage of sites with plaque [23].
- Full-Mouth Bleeding Score (FMBS) representing the percentage of sites showing bleeding on probing [24].
- Probing depth (PD), vertical distance measured from the gingival margin to the bottom of the pocket.
- Clinical attachment level (CAL), vertical distance measured from the CEJ to the bottom of the pocket.
- Gingival recession (GR), vertical distance measured from the CEJ to the gingival margin.
- CEJ-BD, intra-surgical vertical distance measured from the CEJ to the bottom of the intrabony defect (BD).
- INTRA, intra-surgical vertical distance measured from the bone crest to the bottom of the intrabony defect.
- WIDTH, intra-surgical horizontal distance measured from the root surface to the bone crest.

Composite outcome measure (COM) was considered as the combination of two clinical parameters (CAL and PD). Based on a previous study [18] intrabony defects were classified as COM1 (i.e. CAL gain ≥ 3 mm, PD ≤ 4 mm), COM2 (i.e. CAL gain < 3 mm, PD ≤ 4 mm), COM3 (CAL gain ≥ 3 mm, PD > 4 mm), COM4 (CAL gain < 3 mm, PD > 4 mm). The COM was considered as primary outcome.

In addition, gender, age, smoking status, periodontal diagnosis, location of teeth, defects’ morphology (i.e. number of bony walls), surgical flap design and regenerative procedures applied were also recorded. All clinical parameters were assessed using a graduated manual periodontal probe (PCP-UNC 15®,Hu-Friedy, Chicago, IL, USA) at baseline, 1-and 7-years follow-up. The clinical and intra-surgical variables (i.e. CAL, PD, CEJ-BD and INTRA) were assessed at the deepest site of intrabony defects, while FMPS and FMBS were recorded at 6 sites per tooth.

### Clinical procedure

Before periodontal regeneration, patients received non-surgical periodontal treatment with motivation, oral hygiene instruction, risk factors control, supragingival professional mechanical plaque removal (PMPR) and subgingival instrumentation. The non-surgical periodontal therapy based on a quadrant-wise approach was performed by an expert operator (V.I.S.) using a combination of ultrasonic instruments (Instrument PS©EMS Electro Medical System S.A. Nyon, Switzerland) and Gracey metal curettes. (Hu-Friedy,Chicago,IL,USA) [22]. Teeth with increased mobility were splinted before the surgical interventions.

Based on the depth of radiographic intrabony component evaluated using a periapical intraoral radiograph, different surgical flap designs were performed to access the interdental area. Whenever the defects involved one or two sides of a root and it was cleansable from the buccal aspect, the modified minimally invasive surgical technique (M-MIST) was made [25]. If the intrabony defects were not cleansable from the buccal aspect, a minimally invasive surgical technique (MIST) was selected [26]. On the contrary, severe intrabony defects (i.e. extending to mid-third of the root and beyond) were treated using and extended flap [27] with modified papilla preservation technique (SPPT) [28]. After surgical access, the granulation tissue was removed, and the root surface was mechanically treated using ultrasonic scaler and Gracey metal curettes (Hu-Friedy,Chicago,IL,USA). The intrabony defects were approached with different regenerative approaches such as enamel matrix protein derivative alone (EMD), deproteinized bovine bone mineral (DBBM) combined with EMD, or guided tissue regeneration (GTR) using collagen membrane (CM) and DBBM.

The intrabony defects treated with EMD alone or EMD + DBBM received a root conditioning with 24% EDTA gel [29]. Primary wound closure was achieved using a 5–0 or 6–0 monofilament suture material (i.e., ePTFE or Polypropylene) [30]. Post-operative pain was controlled with 600 mg ibuprofen immediately before surgical intervention and after 4 h [31]. No systemic antibiotics were prescribed. All patients were asked to rinse twice daily with a 0.12% chlorhexidine solution [32]. Sutures were removed after 1 week [33] and patients were recalled at 1-,3-,6- and 12-months following treatment for oral hygiene reinforcement, motivation and supragingival plaque removal [34]. The final examination was performed at 12 months. All patients were enrolled in a personalized SPC program for professional oral hygiene.

### Statistical analysis

All clinical variables (i.e. PD, CAL, GR, CEJ-BD, INTRA, WIDTH) were expressed in millimeters, while FMPS and FMBS were reported as percentages. For the parameters gender, smoking habit, tooth location, defect’s configuration, the number and percentage were reported. Descriptive statistics (i.e. mean ± standard deviation) were used to present the clinical results. Since some patients contributed with multiple intrabony defects to the study, the patient was considered as the statistical unit. A patient-based analysis was performed to compare FMPS, FMBS, PD, CAL, and GR between baseline, 1- and 7-years follow-up using a non-parametric statistical test for paired data (i.e. Friedman’s test and Wilcoxon’s test). In addition, a site-level analysis was made. A Chi-square test was used to compare the differences in gender, smoking habit, tooth location, defect’s configuration, type of surgery and type of regenerative procedures, with respect to COM groups at 1-and 7-years follow-up. The evaluation of mean age, PD, CAL, CEJ-BD, INTRA, and WIDTH with respect to the COM groups were made by means of Kruskal–Wallis and Mann–Whitney U test. A *p*-value < 0.05 was considered statistically significant. Treatment success was defined as a site displaying a CAL gain ≥ 3 mm and a PD ≤ 4 mm (COM 1), while the sites displaying the characteristics of COM 2, COM 3 and COM 4 were considered as treatment failure.

The statistical analysis was performed using a statistical software package (IBM-SPSS, IBM Inc.).

## Results

### Demographic characteristics

The characteristics of the participants are summarized in Table 1. A total of 29 periodontal intrabony defects treated in 19 patients (8 males and 11 females) with a mean age 43.9 ± 9.7 years were selected for the analysis. Four patients were tobacco smokers (≤ 10 cigarettes/die). Seventeen patients were affected by generalized stage III/grade C periodontitis, while in 2 patients a molar/incisor pattern stage III/grade C was diagnosed. The majority of the enrolled teeth were located in upper and posterior arch. Eighteen defects showed a residual 3-walls intrabony component, while a residual 2- and 1-wall component was found in 4 and 7 defects, respectively. Twenty-three intrabony defects were treated using an extended flap with SPPT, while 6 defects were accessed using a minimally invasive surgical approach (i.e., MIST or M-MIST). In 7 defects the periodontal regeneration was made using DBBM covered with collagen membrane (i.e. GTR), 8 intrabony defects were treated with EMD alone, while a combination of DBBM and EMD was applied in 14 defects.

**Table 1:** Patients, teeth and defects characteristics

Patient characteristics	Teeth characteristics	Defects characteristics	Surgical flap design	Regenerative Treatment
Number of patients: 19	Number of teeth: 29	Number of defects: 29	Number of defects:29	Number of defects:29
Mean age (y): 43.9 ± 9.7	Anterior/Posterior:16/13	I wall: 7	EF + SPPT: 23	CM + DBBM:7
Range age (y): 14–62	Upper/Lower: 21/8	II walls: 4	MIST: 3	EMD:8
Gender (M/F): 8(M) 11(F)		III walls: 18	M-MIST: 3	EMD + DBBM:14
Smoking habit (S/NS): 4(S) 15(NS)		CEJ-BD (mm):10.3 ± 2.5		
Periodontal diagnosis (N):				
Generalized Stage III/Grade C = 17		BC-BD (mm): 4.9 ± 1.3		
Incisor-molar pattern Stage III/Grade C = 2		WIDTH (mm):4 ± 0.9		

Y = years; M = Male = Female; S = smokers = Non-smokers; N = number; CEJ-BD = Radiographic vertical distance measured from CEJ to the bottom of the defect; BC-BD = Radiographic vertical distance measured from the bone crest to the bottom of the defect; WIDTH = Horizontal distance measured from root surface to the bone crest at the most apical point; EF = Extended flap; SPPT = Simplified Papilla Preservation Technique; MIST = Minimally invasive surgical technique; M-MIST = Modified Minimally Invasive Surgical Technique; CM = Collagen Membrane; DBBM = Deproteinized Bovine Bone Mineral; EMD = Enamel Matrix Derivative

### Patient-level analysis of clinical outcomes

Table 2 illustrated the results of patient-based analysis. FMPS and FMBS significantly increased from baseline to 7- years follow-up (*p* < 0.05). However, FMPS did not change significantly between 1- and 7-years follow-up (*p* > 0.05). At 7 years, FMPS changed from 16.2 ± 3.5% to 20.1 ± 3.5%, while FMBS varied from 12.4 ± 2.7% to 18.7 ± 3.4%. The probing depth (PD) changed significantly at 1 and 7 years (*p* < 0.05). At baseline, the intrabony defects showed a PD of 8.01 ± 1.9 mm, while a PD of 3.96 ± 1.4 mm and 4.42 ± 1.7 mm was detected at 1-, and 7-years follow-up, respectively. The mean clinical attachment level (CAL) varied from 9.43 ± 2.5 mm to 6.22 ± 2.7 mm after 1 year follow-up, whereas a CAL of 7.11 ± 3.1 mm was assessed at 7 years follow-up. Statistically significant differences were found between baseline, 1 year and 7 years (*p* < 0.05). Likewise, the mean of GR changed significantly at the three time points (*p* < 0.05). At 1 year, GR increased from 1.40 ± 1.3 mm to 2.26 ± 1.9 mm, while at 7 years a GR of 2.70 ± 2.0 mm was recorded.

**Table 2:** Patient-level analysis of changes in FMPS,FMBS,PD,CAL and GR at baseline,1- and 7-years follow-up

	**Baseline**	**1 years**	**7 years**	**Δ BL-1y**	**Δ BL-7y**	**Δ 1y-7y**	***p*-value**^**§**^	***p*-value**^**§§**^ **(BL-1y)**	***p*-value**^**§§**^ **(BL-7 y)**	***p*-value**^**§§**^ **(1y-7y)**
FMPS (%)	16.2 ± 3.5	18.9 ± 3.1	20.1 ± 3.5	2.6 ± 2.0	3.8 ± 3.5	1.2 ± 3.4	0.0001(s)*	0.0001(s)*	0.002(s)*	0.083(ns)**
FMBS (%)	12.4 ± 2.7	16.4 ± 3.0	18.7 ± 3.4	4.4 ± 3.0	6.5 ± 3.8	2.1 ± 4.2	0.0001(s)*	0.0001(s)*	0.0001(s)*	0.025(s)*
PD (mm)	8.01 ± 1.9	3.96 ± 1.4	4.42 ± 1.7	3.9 ± 1.4	3.4 ± 1.4	0.4 ± 0.7	0.0001(s)*	0.0001(s)*	0.0001(s)*	0.013(s)*
CAL (mm)	9.43 ± 2.5	6.22 ± 2.7	7.11 ± 3.1	3.2 ± 1.1	2.3 ± 1.4	0.9 ± 0.9	0.0001(s)*	0.0001(s)*	0.0001(s)*	0.0001(s)*
GR (mm)	1.40 ± 1.3	2.26 ± 1.9	2.70 ± 2.0	0.8 ± 1.2	1.2 ± 1.5	0.4 ± 0.8	0.0001(s)*	0.004(s)*	0.0001(s)*	0.004(s)*

FMPS = Full mouth plaque score; FMBS = Full mouth bleeding score; PD = probing depth; CAL = clinical attachment level; GR = gingival recession; BL = baseline; y = year; § Kruskal–Wallis test; §§ Mann–Whitney U test

^*^Statistically significant difference

^**^Non-statistically significant difference

## Population, defects and treatment modalities in composite outcome measure groups

At 1 year following regenerative treatment, 22 intrabony defects were classified as COM1 and 2 defects (1 defect per group) were associated to COM2 and COM3, respectively. Five defects were considered as COM4. No statistically significant differences were recorded between COM groups with respect to age, gender, smoking habit and teeth location (*p* > 0.05).

Considering defect’s morphology (i.e. number of residual bone walls) evaluated during surgical treatment, 2 intrabony defects with one residual wall were classified as COM1, 1 defect as COM3 and 4 defects as COM4. Four intrabony defects with two residual walls were associated at COM1 group. Sixteen three-walls defects were considered COM1, 1 defect COM2 and 1 defect COM4, respectively. Distribution of COMs varies significantly between groups based on number of residual bone walls (*p* < 0.05).

At baseline, CEJ-BD was 9.5 ± 1.8 mm in COM 1 group, 8.0 ± 0.0 mm in COM 2 group, 18.0 ± 0.0 mm in COM 3 group and 12.6 ± 1.7 mm in COM 4 group. Statistically significant differences between groups were found (*p* < 0.05). Likewise, a statistically significant difference (*p* < 0.05) was noted between groups in terms of vertical intra-bony component (INTRA). Defects of COM1 group showed an INTRA of 4.6 ± 1.0 mm, while in COM 2 and COM 3 groups an INTRA of 3.0 ± 0.0 mm and 7.0 ± 0.0 mm were recorded, respectively. Defects classified as COM 4 displayed an INTRA of 6.4 ± 1.1 mm. On the contrary, no statistically significant difference between COM groups were noted for the WIDTH parameter (*p* > 0.05). Defects allocated in COM 1 and COM 4 groups showed a WIDTH of 4.2 ± 0.4 mm, meanwhile the WIDTH of defects of COM 3 and COM 4 groups was 4.0 ± 0.0 mm.

The majority of the defects were accessed using an extended flap, and no statistically significant differences were recorded between groups (*p* > 0.05). The combination of EMD and DBBM was used to treat 11 defects in COM 1 groups, 1 defect in COM 2 and 2 defects in COM 4. The application of EMD alone was performed in 7 defects of COM 1 group, and in 1 defects of COM 4 group. No statistically significant differences were recorded between groups (*p* > 0.05) (TAB.3).

**Table 3:** Population, defects and treatment modalities characteristics in COM groups at 1 year follow-up

		COM1 (*N* = 22)	COM2 (*N* = 1)	COM3 (*N* = 1)	COM4 (*N* = 5)	*p*-value
**Patients**	**AGE (years)**					
	*mean* ± *SD*	41.1 ± 13.1	42 ± 0.0	39 ± 0.0	39,2 ± 15,2	0,911(NS)**
	*Median*	37.3			27	
	*95%CI*	37.3–48.0			27.0–50.5	
	**GENDER**					
	**Male**	6	0	1	3	0.224(NS)**
	**Female**	16	1	0	2	
	**SMOKING HABIT**					
	**Non-smokers**	18	1	0	5	0.688 (NS)**
	**Smokers**	4	0	1	0	
		COM1 (*N* = 22)	COM2 (*N* = 1)	COM3 (*N* = 1)	COM4 (*N* = 5)	*p*-value
Teeth Location	DENTAL ARCH					
	**Upper**	14	1	1	5	0.139(NS)**
	**Lower**	8	0	0	0	
	**TOOTH TYPE**					
	**Anterior**	12	0	1	3	0.553(NS)**
	**Posterior**	10	1	0	2	
		COM1 (*N* = 22)	COM2 (*N* = 1)	COM3 (*N* = 1)	COM4 (*N* = 5)	*p*-value
Defects	**NUMBER OF WALLS**					
	**I wall**	2	0	1	4	0.019(S)*
	**II walls**	4	0	0	0	
	**III walls**	16	1	0	1	
	INTRASURGICAL PARAMETERS					
	CEJ-BD (mm)					
	mean ± SD	9.5 ± 1.8	8.0 ± 0.0	18,0 ± 0.0	12,6 ± 1.7^#^	0.013(S)*
	median	10			13.0	
	95%CI	8–11			11–14	
	INTRA (mm)					
	mean ± SD	4.6 ± 1.0^§^	3.0 ± 0.0	7.0 ± 0.0	6.4 ± 1.1^#^	0.007(S)*
	median	4			6	
	95%CI	4–5			5.5–7-5	
	WIDTH (mm)					
	mean ± SD	4.2 ± 0.4^§^	4.0 ± 0.0	4.0 ± 0.0	4.2 ± 0.4^#^	0.915 (NS)**
	median	4			4	
	95%CI	4.0–4.5			4.0–4.5	
		COM1 (*N* = 22)	COM2 (*N* = 1)	COM3 (*N* = 1)	COM4 (*N* = 5)	*p*-value
Surgical treatment	SURGICAL FLAP DESIGN					
	EF + SPPT	16	1	1	5	0.492(NS)**
	MIST	3	0	0	0	
	M-MIST	3	0	0	0	
	REGENERATIVE PROCEDURE					
	CM + DBBM	4	0	1	2	0.495(NS)**
	EMD	7	0	0	1	
	EMD + DBBM	11	1	0	2	

CI = Confidence Interval; SD = Standard deviation; CEJ-BD = Intrasurgical vertical distance measured from CEJ to the bottom of the defect; BC-BD = Intrasurgical vertical distance measured from the bone crest to the bottom of the defect; WIDTH = Intrasurgical horizontal distance measured from root surface to the bone crest at the most apical point; EF = Extended flap; SPPT = Simplified Papilla Preservation Technique; MIST = Minimally invasive surgical technique; M-MIST = Modified Minimally Invasive Surgical Technique; CM = Collagen Membrane; DBBM = Deproteinized Bovine Bone Mineral; EMD = Enamel Matrix Derivative; § = comparison between COM groups;* Statistically significant difference;**Non-statistically significant difference; #Statistically significant difference vs COM1; †Statistically significant difference vs COM2; ‡ Statistically significant difference vs COM3; § Statistically significant difference vs COM4. COM = Composite outcome measure

The distribution of patients, defects and treatment modalities after 7 years follow-up is summarized in Table 4. At 7 years, the patient distribution allocated in COM1, COM2, COM3 and COM4 related to age, gender, smoking habit, and teeth location did not show statistically significant differences (*p* > 0.05). On the contrary, the difference of defect distribution in COM groups related to residual bone walls, CEJ-BD, INTRA and WIDTH was statistically significant (*p* < 0.05). Furthermore, no statistically significant difference was recorded between COM groups in terms of surgical flap design and regenerative procedures applied (*p* > 0.05) (TAB.4).

**Table 4:** Population, defects and treatment modalities characteristics in COM groups at 7 years follow-up

		COM1 (*N* = 13)	COM2 (*N* = 8)	COM3 (*N* = 0)	COM4 (*N* = 8)	*p*-value
**Patients**	**AGE (years)**					
	*mean* ± *SD*	39.9 ± 16.9	42.4 ± 4.3	0.0 ± 0.0	40,5 ± 11,8	0,945(NS)**
	*Median*	45.0	42		41.5	
	*95%CI*	24.5–51.5	38.5–45.0		39.3–47	
	**GENDER**					
	**Male**	4	1	0	5	0.102(NS)**
	**Female**	9	7	0	3	
	**SMOKING HABIT**					
	**Non-smokers**	11	7	0	7	0.975 (NS)**
	**Smokers**	4	0	1	0	
		COM1 (*N* = 13)	COM2 (*N* = 8)	COM3 (*N* = 0)	COM4 (*N* = 8)	*p*-value
Teeth Location	DENTAL ARCH					
	**Upper**	7	1	1	5	0.131(NS)**
	**Lower**	6	0	0	0	
	**TOOTH TYPE**					
	**Anterior**	12	4	0	5	0.874(NS)**
	**Posterior**	10	1	0	2	
		COM1 (*N* = 13)	COM2 (*N* = 8)	COM3 (*N* = 0)	COM4 (*N* = 8)	*p*-value
Defects	**NUMBER OF WALLS**					
	**I wall**	0	1	0	6	0.002(S)*
	**II walls**	3	1	0	0	
	**III walls**	10	6	0	2	
	INTRASURGICAL PARAMETERS					
	CEJ-BD (mm)					
	mean ± SD	9.2 ± 1.4^§^	9.4 ± 2.3^§^	0.0 ± 0.0	12,9 ± 2.5^#†^	0.004(S)*
	median	10	8.5		12.5	
	95%CI	8–10	11.8		11–14	
	INTRA (mm)					
	mean ± SD	4.0 ± 1.0^§^	4.0 ± 0.8^§^	0.0 ± 0.0	6.0 ± 1.3^#†^	0.009(S)*
	median	4	4		6	
	95%CI	5–5.5	3.3–4.8		5–7	
	WIDTH (mm)					
	mean ± SD	4.4 ± 0.7^§†^	3.1 ± 0.8^#^	0.0 ± 0.0	4.1 ± 0.6^†^	0.005 (S)**
	median	4	3		4	
	95%CI	4.0–4.5	2.3–4		4.0–4.8	
		COM1 (*N* = 22)	COM2 (*N* = 1)	COM3 (*N* = 1)	COM4 (*N* = 5)	*p*-value
Surgical treatment	SURGICAL FLAP DESIGN					
	EF + SPPT	10	5	0	8	0.173(NS)**
	MIST	2	1	0	0	
	M-MIST	1	2	0	0	
	REGENERATIVE PROCEDURE					
	CM + DBBM	2	1	0	4	0.105(NS)**
	EMD	7	0	0	1	
	EMD + DBBM	5	6	0	3	

CI = Confidence Interval; SD = Standard deviation; CEJ-BD = Intrasurgical vertical distance measured from CEJ to the bottom of the defect; BC-BD =

Intrasurgical vertical distance measured from the bone crest to the bottom of the defect; WIDTH = Intrasurgical horizontal distance measured from root

surface to the bone crest at the most apical point; EF = Extended flap; SPPT = Simplified Papilla Preservation Technique; MIST = Minimally invasive surgical

technique; M-MIST = Modified Minimally Invasive Surgical Technique; CM = Collagen Membrane; DBBM = Deproteinized Bovine Bone Mineral; EMD =

Enamel Matrix Derivative; § = comparison between COM groups;* Statistically significant difference;**Non-statistically significant difference; #Statistically

significant difference vs COM1; †Statistically significant difference vs COM2; ‡ Statistically significant difference vs COM3; § Statistically significant

difference vs COM4; COM = Composite outcome measure

## PD and CAL changes in composite outcome measure groups at 1-, and 7-years follow-up

Clinical parameters (i.e. PD and CAL) in COM1, COM2, COM3 and COM4 are illustrated in Table 5. At 7 years, PD and CAL of defects allocated in COM1 changed significantly from 7.07 ± 0.0 mm to 3.6 ± 0.8 mm and from 8.2 ± 1.2 mm to 5.5 ± 1.6 mm, respectively (*p* < 0.05). One defect with a PD of 6.0 ± 0.0 mm and CAL of 8.0 ± 0.0 mm was allocated in COM2 group. After 7 years follow-up, the PD was 4.0 ± 0.0 mm and a CAL of 7.0 ± 0.0 mm was recorded. Also in COM 3 group,1 defect was found. PD and CAL varied from 13.0 ± 0.0 mm and 16.0 ± 0.0 mm to 6.0 ± 0.0 mm for each parameter. At baseline, intrabony defects classified as COM 4 showed a PD of 10.0 ± 1.2 mm, while at 1 year the PD was 6.0 ± 1.2 mm, and after 7 years a PD of 7.0 ± 1.2 mm was assessed. Statistically significant difference was recorded in PD change between baseline and 1 year follow-up (*p* < 0.05), while no statistically significant changes were found between 1 and 7 years follow-up (*p* > 0.05). Likewise, CAL statistically change from baseline to 1 year follow-up (*p* < 0.05), and no statistically significant differences were recorded from 1 and 7 years follow-up. At baseline and after 1 year follow-up, the CAL was 11.4 ± 2.1 mm and 9.6 ± 2.2 mm respectively, while a CAL of 11.0 ± 2.9 mm was observed after 7 years follow-up (Table 5).

**Table 5:** Probing depth (PD) and Clinical attachment level (CAL) in COM groups at baseline, 1-and 7-years follow-up

		**Baseline**	**1 years**	**7 years**	**Δ 1y-7y**	***p*-value** **(BL-1y)**	***p*-value (1y-7y)**
**COM1**	**PD (mm)**						
(*N* = 22)	mean ± SD	7.0 ± 0.7	3.3 ± 0.7	3.6 ± 0.8	0.3 ± 0.6	0.001(S)*	0.035(S)*
	median	7	3	4	0		
	95%CI	7.0–8.0	3.0–4.0	3.0–5-0	0–1		
	**CAL (mm)**						
	mean ± SD	8.2 ± 1.2	4.7 ± 1.3	5.5 ± 1.6	0.8 ± 0.9	0.001(S)*	0.002(S)*
	median	8	5	5	1.0		
	95%CI	8.0–9.0	4.0–6.0	4.8–7.0	0		
**COM2**	**PD (mm)**						
(*N* = 1)	mean ± SD	6.0 ± 0.0	4.0 ± 0.0	4.0 ± 0.0	0		
	median						
	95%CI						
	**CAL (mm)**						
	mean ± SD	8.0 ± 0.0	7.0 ± 0.0	7.0 ± 0.0	0		
	median						
	95%CI						
**COM3**	**PD (mm)**						
(*N* = 1)	mean ± SD	13.0 ± 0.0	5.0 ± 0.0	6.0 ± 0.0	1		
	median						
	95%CI						
	**CAL (mm)**						
	mean ± SD	16.0 ± 0.0	11.0 ± 0.0	6.0 ± 0.0	1		
	median						
	95%CI						
**COM4**	**PD (mm)**						
(*N* = *5*)	mean ± SD	10.0 ± 1.2	6.0 ± 1.2	7.0 ± 1.2	1.0 ± 0.7	0.041(S)*	0.059 (NS)**
	median	10	6	7	2		
	95%CI	9.0–11	5.0–7.0	6.0–8.0	0.5–1.5		
	**CAL (mm)**						
	mean ± SD	11.4 ± 2.1	9.6 ± 2.2	11.0 ± 2.9	1.4 ± 0.9	0.034(S)*	0.059 (NS)**
	median	12	11	12	2		
	95%CI	9.5–13.0	7.5–11	8.5–13	0.5–2		

CI = Confidence Interval; SD = Standard deviation; * Statistically significant difference;**Non-statistically significant difference; COM = Composite outcome measure

### Treatment success and failure

Treatment success (i.e. COM1) and treatment failure (i.e. COM2, COM3, COM4) are illustrated in Table 6. In addition, the changes in defect allocation in COM groups between 1 and 7 years follow-up were also reported. After 1 year follow-up treatment success (i.e. COM1) was achieved in 22 defects (75.9%), while 1 defect (3.4%) was allocated in COM2 and 1 (3.4%) in COM 3 group. The residual 5 defects (17.2%) were classified as COM4.

**Table 6:** Treatment success, failure and COM groups changes of intrabony defects at 1- and 7-years follow-ups

	**Treatment success** (N/%)	**Treatment Failure** (N/%)		
	**(COM1)**	**(COM2)**	(**COM3)**	**(COM4)**
**1 year follow-up**	22(75.9)	1(3.4)	1(3.4)	5(17.2)
**7 years follow-up**	13(44.8)	8(27.6)	0	8(27.6)
	**1 year follow-up** **(N/%)**		**7 years follow-up** **(N/%)**	
**COM1 (**N/%)	22 (75.9)	**COM 1** (N/%)	13(44.8)	
		**COM 2** (N/%)	7(24.1)	
		**COM 3** (N/%)	0	
		**COM 4** (N/%)	2(6.9)	
**COM2 (**N/%)	1(3.4)	**COM 1** (N/%)	0	
		**COM 2** (N/%)	1(3.4)	
		**COM 3** (N/%)	0	
		**COM 4** (N/%)	0	
**COM3 (**N/%)	1(3.4)	**COM 1** (N/%)	0	
		**COM 2** (N/%)	0	
		**COM 3** (N/%)	0	
		**COM 4** (N/%)	1(3.4)	
**COM4** (N/%)	5(17.2)	**COM 1** (N/%)	0	
		**COM 2** (N/%)	0	
		**COM 3** (N/%)	0	
		**COM 4** (N/%)	5(17.2)	

*N* = number of intrabony defects; % = Percentage of intrabony defects; * Statistically significant difference;**Non-statistically significant difference; COM = Composite outcome measure

At 7 years, the percentage of treatment success decreased to 44.8% (i.e. 13 defects). Eight intrabony defects (27.6%) were assessed as COM2 and 8 (27.6%) as COM4, while no defects were allocated in COM3 group. After 7 years, out of the 22 defects (75.9%) allocated in the COM1, 13 defects (44.8%) were stable, while 7 (24.1%) defects were allocated in COM2, and 2 defects (6.9%) in COM4. No change in COM group was observed for the defect classified as COM2, while the defect allocated in COM3 group was assigned at COM4 group. All defects of clinical COM 4 did not change COM group (TAB.6).

## Discussion

The present retrospective study evaluated the long-term results of intrabony defects treated with regenerative therapy using a composite outcome measure (COM).

The findings suggested that the clinical benefits achieved following periodontal regeneration treatment can be maintained over a period of 7 years. Similar results were presented in previous investigations based on long term evaluations of intrabony defects treated using different regenerative approaches [34–36].

At 1-, and 7-years, all clinical parameters improved significantly with respect to baseline, however the comparison between 1-, and 7-years follow-up showed a slight worsening.

At 7 years, a PD increase of 0.4 mm and CAL loss of 0.9 mm were observed with respect to 1 year follow-up. These findings agree to those reported by Sculean and co-workers [37], who assessed a statistically significant increase in PD between 1 year and 9-year follow-up. Potential explanation may be related to the presence of residual pockets (PD > 4 mm) in 6 sites after regeneration treatment (e.g. at 1 year follow-up). This aspect is in agreement with a retrospective study evaluating the impact of residual pockets on periodontitis progression in patients enrolled in supportive periodontal therapy. In fact, the presence of residual pocket (PD > 4 mm) after completion of periodontal therapy represented a risk factor for disease progression [38].

In contrast with previous results [20], treatment success (i.e., COM1) was detected in 75.9% of the defects after 1 year follow-up, while Simonelli and co-workers reported a treatment success of 90.5% at 6 months. Probably, the lower percentage of treatment success achieved in the current analysis depends on the different definition of treatment success applied, since the authors considered successfully treated the defects allocated in COM1, COM2, and COM3. On the contrary, in the current study treatment success was defined as a site displayed a CAL gain ≥ 3 mm and a PD ≤ 4 mm (i.e., COM 1), since at least 3 mm of CAL gain was considered clinically relevant to detect the efficacy of regenerative therapy [39] and a residual pocket ≤ 4 mm is not considered a risk factor for the tooth prognosis. In both studies, the lack of information on the depth of residual pockets and the small number of defects allocated in each COM group does not allow to draw any substantial conclusions on the present topic.

At 7 years, the treatment success (44.8%) was similar to those reported by De Ry and co-workers [40] who observed an overall treatment success of 42.6% after a mean of 10 years follow-up.

The use of different regenerative modalities (i.e. GTR, EMD and EMD + DBBM) as well as different surgical accesses (i.e. MIST, M-MIST and extended flap with papilla preservation) may be considered a bias because a wide heterogeneity in terms of clinical results could be determined [41]. However, each intrabony defect received the proper regenerative procedure based on the number of residual bone walls following the current evidence [27]. In other words, contained defects were treated using EMD alone [42], while in non-contained defects resorbable membranes and DBBM (i.e., GTR) or EMD combined with DBBM were applied [43], since EMD alone appeared to yield less PD reduction and CAL gain compared to GTR in the treatment of non-contained defects [44].

In the present analysis single-rooted and multi-rooted teeth were included. This fact did not represent a bias, since the multi-rooted teeth with furcation defects were excluded.

Since the present retrospective analysis involved patients treated in routine clinical practice, defects affecting two adjacent teeth (i.e., ramps and planes) did not receive periodontal regeneration. These defects resulted in a wound with a reduced blood supply and the periodontal regeneration was not considered predictable. Ramps are characterized by different apico-coronal levels of the residual buccal and lingual bone, while planes occur when the bone loss of interdental space is horizontal, but the middle-buccal bone and/or middle-buccal bone of adjacent teeth is preserved more coronally [45].

Likewise, craters (i.e., intrabony defects affecting two adjacent teeth with a dip interproximal bone crest confined within the buccal and oral walls of two adjacent root surfaces, both presenting with similar attachment loss) present a reduced vascular supply [45]. These defects were treated by means of periodontal regeneration since the craters reflect the anatomy of the bone in the interdental area as a contained concave ridge with a potential capacity for the regeneration. However, data of a systematic review [46] reported that no meaningful conclusion can be draw on the regenerative potential of craters.

Although data of a systematic reviews [8, 47] on preclinical studies showed that most biomaterials and biologic agents used either alone or in various combination promoted periodontal regeneration, the intrabony defects were treated using only these three regenerative procedures (i.e., GTR, EMD + DBBM or EMD alone) as well as recommended by the EFP (European Federation of Periodontology) clinical practice guidelines [25].

The majority of the defects were accessed by means of an extended flap with papilla preservation technique, while only 6 defects were treated using a minimally invasive surgical approach (i.e. MIST and M-MIST). This fact did not influence the final outcomes, since data from a previous study [48] reported that MIST or M-MIST did not show significant differences in probing parameters with respect to extended flap with papilla preservation.

### Limitations

Some patients contributed with multiple defects, and a patient-based analysis was performed for the probing parameters. However, the primary outcome is a site-related categorical variable, hence patient-specific bias could not be ruled out.

The small sample represents a limitation of the study, especially in the allocation of intrabony defects into COM groups. This aspect limited the possibility to detect significant conclusions of the predictive value of COM on long term clinical results of periodontal regeneration. However, the purpose of this study was not to test the prognostic value of COM of intrabony defects enrolled in SPC following periodontal regeneration, but to evaluate the long- term outcomes of intrabony defects treated with a regenerative approach using a combination of two endpoints (i.e. CAL gain and PD reduction) included in a composite outcome measure (COM). Another limitation is the lack of examiner standardization and calibration due to the retrospective nature of the study. Since a previous study [19] assessed the efficacy of periodontal regeneration using a combination of two endpoints (i.e. CAL gain and radiographic defect fill) included in a composite outcome measure (COM), the lack of radiographic evaluation could be considered a limitation of the study. However, the purpose of the present study was to evaluate the long-term results of intrabony defects following periodontal regenerative therapy using simplified COM [18]. Simplified COM evaluates the effect of periodontal regenerative therapy basing exclusively on clinical measurements (i.e. CAL gain and PD reduction). For these reasons, the radiographic analysis was not reported.

## Conclusion

Within their limitations, the results of the present study indicated that the clinical improvements obtained following regenerative therapy can be maintained over a period of 7 years. However, treatment success (i.e. COM1) was not always achieved.
